# Global Health research abstracts: June ‘23

**DOI:** 10.1016/j.afjem.2023.05.006

**Published:** 2023-06-06

**Authors:** Dr. Jonathan Kajjimu

**Affiliations:** Faculty of Medicine, Mbarara University of Science and Technology, Mbarara, Uganda

## Abstract

The African Journal of Emergency Medicine, in partnership with several other regional emergency medicine journals, publishes abstracts from each respective journal. Abstracts are not necessarily linked to open access papers however, all abstracts are accessible without subscription.

**Annals of Emergency Medicine (North America)**

**Occupational Accidents Among Search and Rescue Providers During Mountain Rescue Operations and Training Events.**

Milani, M., Roveri, G., Falla, M., Dal Cappello, T., & Strapazzon, G. (2023). *Annals of Emergency Medicine*.

DOI: https://doi.org/10.1016/j.annemergmed.2022.12.015

**Study objective**

We analyzed occupational accidents reported among Corpo Nazionale Soccorso Alpino e Speleologico (CNSAS) providers during mountain search and rescue operations and training events in Italy (1999 to 2019).

**Methods**

We extracted anonymized data from the CNSAS accident database for all cases of injured mountain search and rescue providers that activated CNSAS insurance (1999 to 2019). We report epidemiological characteristics, mechanisms, type, and severity of injury or illness, clinical outcome, and recovery time.

**Results**

A total of 784 cases of injuries in CNSAS mountain search and rescue providers were recorded. Forty-one percent of the cases occurred during rescue operations and 59% during training events. Overall, trauma was the main cause of injury (96%), whereas only 4% of the cases were classified as medical or environmental illnesses. Moderate injury (National Advisory Committee for Aeronautics II to III) occurred in 80% of the reported accidents. Recovery time differed based on the degree of accident severity. Fatalities occurred in 2% of the cases reported and occurred during rescue operations only.

**Conclusion**

In this long-term retrospective analysis, we showed that accidents occurred among mountain search and rescue providers both during rescue operations and training events. Given the high prevalence and associated costs, it is of pivotal importance to understand the epidemiology and characteristics of occupational injury and illness among this out-of-hospital workforce to better inform future prevention strategies.

Reproduced with permission.

**Canadian Journal of Emergency Medicine (North America)**

**Alternative care models for paramedic patients from long-term care centers: a national survey of Canadian paramedic services.**

Leduc, S., Wells, G., Thiruganasambandamoorthy, V., Kelly, P., & Vaillancourt, C. (2023). *Canadian Journal of Emergency Medicine*, 1-9.

DOI: https://doi.org/10.1007/s43678-023-00471-9

**Introduction**

Long-term care (LTC) patients do poorly when transported to emergency departments (ED). Community paramedic programs deliver enhanced care in their place of residence, yet few programs are reported in the literature. We conducted a national cross-sectional survey of land ambulance services to understand if such programs exist in Canada, and what the perceived needs and priorities are for future programs.

**Methods**

We emailed a 46 question survey to paramedic services across Canada. We asked about service characteristics, current ED diversion programs, existing diversion programs specific to LTC patients, priorities for future programs, the potential impact of such programs, and what the feasibility and barriers are to implementing programs that treat LTC patients on-site, avoiding an ED visit.

**Results**

We received responses from 50 sites across Canada, providing services to 73.5% of the total population. Almost a third (30.0%) had existing treat-and-refer programs, and 65.5% of services transported to destinations other than an ED. Almost all respondents (98.0%) felt the need for programs to treat LTC patients on-site, and 36.0% had existing programs. The top priorities for future programs were support for patients being discharged (30.6%), extended care paramedics (24.5%), and respiratory illness treat-in-place programs (20.4%). The highest potential impact was expected from support for patients being discharged (62.0%) and respiratory illness treat-in-place programs (54.0%). Required changes in legislation (36.0%) and required changes to the system of medical oversight (34.0%) were identified as top barriers to implementing such programs.

**Conclusion**

There is a significant mismatch between the perceived need for community paramedic programs treating LTC patients on-site, and the number of programs in place. Programs could benefit from standardized outcome measurement and the publication of peer-reviewed evidence to guide future programs. Changes in legislation and medical oversight are needed to address the identified barriers to program implementation.

Reproduced with permission.

**Emergencias (Europe)**

**Performance of 3 functional scales for predicting adverse outcomes at 30 days in older patients discharged from emergency departments.**

Fernández Alonso C, Del Arco Galán C, Torres Garate R, Madrigal Valdés JF, Romero Pareja R, Bibiano Guillén C, et al.  *Emergencias. In press. DOI: 885.*

https://bit.ly/3Lt3ckx

**Objective**

To compare the ability of 3 functional scales (the Clinical Functional Scale [CFS], the Functional Index – eMergency [FIM], and the Identification of Seniors at Risk [ISAR] scale) to predict adverse outcomes at 30 days in older patients discharged from hospital emergency departments (EDs).

**Methods**

Secondary analysis of data from the FRAIL-Madrid registry of patients aged 75 years or older who were discharged from Madrid EDs over a period of 3 months in 2018 and 2019. Functional was defined by a CFS score over 4, a FIM score over 2, or an ISAR score over 3. The outcome variables were revisits to an ED, hospitalization, functional decline, death, and a composite variable of finding any of the previously named variables within 30 days of discharge.

**Results**

A total of 619 patients were studied. The mean (SD) age was 84 (7) years, and 59.1% were women. The CFS identified as frail a total of 339 patients (54.8%), the FIM 386 (62.4%), and the ISAR 301 (48.6%). An adverse outcome occurred within 30 days in 226 patients (36.5%): 21.5% revisited, 12.6% were hospitalized, 18.4% experienced functional decline, and 3.6% died. The areas under the receiver operating characteristic curves were as follows: CFS, 0.66 (95% CI, 0.62-0.70; P = .022); FIM, 0.67 (95% CI, 0.62-0.71; P = .021), and ISAR, 0.64 (95% CI, 0.60-0.69; P = .023). Adjusted odds ratios (aOR) showed that frailty was an independent risk factor for presenting any of the named adverse outcomes: aOR for CFS >4, 3.18 (95% CI, 2.02-5.01), P < .001; aOR for FIM > 2, 3.49 (95% CI, 2.15-5.66), P < .001; and aOR for ISAR >3, 2.46 (95% CI, 1.60-3.79), P < .001.

**Conclusions**

All 3 scales studied — the CFS, the FIM and the ISAR — are useful for identifying frail older patients at high risk of developing an adverse outcome (death, functional decline, hospitalization, or revisiting the ED) within 30 days after discharge.

Reproduced with permission.

**Emergency Medicine Journal (Europe)**

**Evaluating the impact of a pulse oximetry remote monitoring programme on mortality and healthcare utilisation in patients with COVID-19 assessed in Emergency Departments in England: a retrospective matched cohort study.**

Beaney, T., Clarke, J., Alboksmaty, A., Flott, K., Fowler, A., Benger, J., ... & Neves, A. L. (2023). *Emergency Medicine Journal*.

DOI: http://dx.doi.org/10.1136/emermed-2022-212377

**Background**

To identify the impact of enrolment onto a national pulse oximetry remote monitoring programme for COVID-19 (COVID-19 Oximetry @home; CO@h) on health service use and mortality in patients attending Emergency Departments (EDs).

**Methods**

We conducted a retrospective matched cohort study of patients enrolled onto the CO@h pathway from EDs in England. We included all patients with a positive COVID-19 test from 1 October 2020 to 3 May 2021 who attended ED from 3 days before to 10 days after the date of the test. All patients who were admitted or died on the same or following day to the first ED attendance within the time window were excluded. In the primary analysis, participants enrolled onto CO@h were matched using demographic and clinical criteria to participants who were not enrolled. Five outcome measures were examined within 28 days of first ED attendance: (1) Death from any cause; (2) Any subsequent ED attendance; (3) Any emergency hospital admission; (4) Critical care admission; and (5) Length of stay.

**Results**

15, 621 participants were included in the primary analysis, of whom 639 were enrolled onto CO@h and 14 982 were controls. Odds of death were 52% lower in those enrolled (95% CI 7% to 75%) compared with those not enrolled onto CO@h. Odds of any ED attendance or admission were 37% (95% CI 16% to 63%) and 59% (95% CI 32% to 91%) higher, respectively, in those enrolled. Of those admitted, those enrolled had 53% (95% CI 7% to 76%) lower odds of critical care admission. There was no significant impact on length of stay.

**Conclusions**

These findings indicate that for patients assessed in ED, pulse oximetry remote monitoring may be a clinically effective and safe model for early detection of hypoxia and escalation. However, possible selection biases might limit the generalisability to other populations.

Reproduced with permission.

**Hong Kong Journal of Emergency Medicine (Asia)**

**Comparison of fibre-optic-guided endotracheal intubation through a supraglottic airway device versus hyperangulated video laryngoscopy by emergency physicians: A randomised controlled study in cadavers.**

Groombridge, C. J., Maini, A., Mathew, J., Fritz, P., Kim, Y., Fitzgerald, M., ... & O'Reilly, G. (2021). *Hong Kong Journal of Emergency Medicine*, 10249079211034272.

DOI: https://doi.org/10.1177/10249079211034272

**Background**

After failed endotracheal intubation, using direct laryngoscopy, rescued using a supraglottic airway device, the choice of subsequent method to secure a definitive airway is not clearly determined.

**Objective**

The aim of this study was to compare the time to intubation using a fibre-optic airway scope, to guide an endotracheal tube through the supraglottic airway device, with a more conventional approach using a hyperangulated video laryngoscope.

**Methods**

A single-centre randomised controlled trial was undertaken. The population studied were emergency physicians working in an adult major trauma centre. The intervention was intubation through a supraglottic airway device guided by a fibre-optic airway scope. The comparison was intubation using a hyperangulated video laryngoscope. The primary outcome was time to intubation. The trial was registered with ANZCTR.org.au (ACTRN12621000018819).

**Results**

Four emergency physicians completed intubations using both of the two airway devices on four cadavers for a total of 32 experiments. The mean time to intubation was 14.0 s (95% confidence interval = 11.1–16.8) in the hyperangulated video laryngoscope group compared with 29.2 s (95% confidence interval = 20.7–37.7) in the fibre-optic airway scope group; a difference of 15.2 s (95% confidence interval = 8.7–21.7, *p* < 0.001). All intubations were completed within 2 min, and there were no equipment failures or evidence of airway trauma.

**Conclusion**

Successful intubation of the trachea without airway trauma by emergency physicians in cadavers is achievable by either fibre-optic airway scope via a supraglottic airway device or hyperangulated video laryngoscope. Hyperangulated video laryngoscope was *statistically* but arguably not *clinically* significantly faster than fibre-optic airway scope via supraglottic airway device.

Reproduced with permission.

